# Improving Collaboration Between Staff, Family Members, and Artists in Long-Term Dementia Care: A Participatory Action Research Project Into Health Care Clowning

**DOI:** 10.1177/10497323251316426

**Published:** 2025-03-04

**Authors:** Lieke de Kock, Barbara Groot, Jolanda Lindenberg, Charlotte Langemeijer, Silvia de Faveri, Katharina Lessiak, Elisabeth Fajt, Carmen Valero, Tineke A. Abma

**Affiliations:** 1 4496 Leiden University Medical Centre, Leiden, Netherlands; 2 Leyden Academy on Vitality and Ageing, Leiden, Netherlands; 3 1190 Vrije Universiteit Amsterdam, Amsterdam, Netherlands; 4 576328 CliniClowns, Amersfoort, Netherlands; 5 RED NOSES International, Vienna, Austria; 6 ROTE NASEN Austria, Vienna, Austria; 7 ROTE NASEN Germany, Berlin, Germany

**Keywords:** dementia care, arts-based interventions, participatory action research, health care clowning

## Abstract

A growing amount of evidence shows the positive impact of arts-based interventions in dementia care. Existing studies focus on the impact of such interventions on individuals with dementia, yet there is little known about contextual factors influencing the impact of such practices. Contextual factors include personal and relational processes, such as the collaboration between staff, family members, and artists. It also includes making specific organizational choices about the way in which arts and care organizations structure and organize their collaboration. The study aimed to investigate contextual factors influencing the potential impact of health care clowning for persons with dementia. Through multi-country participatory action research (PAR) into health care clowning in dementia care, this study engaged artists (health care clowns), staff, family members, and representatives from four long-term dementia care facilities and three health care clowning organizations. The presented findings show that for arts-based interventions to have sustainable impact within the context of long-term dementia care, focusing on the intervention itself is not enough. Additional time and space are needed for implementation of the intervention and good collaboration on the work floor. The results of this study demonstrate that elements in the PAR process such as open dialogue and arts-based research methods can create communicative spaces which can serve as a catalyst for effective implementation of arts-based practices in long-term dementia care. Elements of the PAR process can therefore be regarded as a form of successful boundary work and in the future could be applied when implementing arts-based interventions in care settings.

## Introduction

An increasing number of individuals are diagnosed with dementia worldwide, making it important to provide care for them and focus on the well-being of this group and their relatives (World Health Organisation, 2017). There is a growing body of evidence supporting the positive impact of arts-based interventions on the well-being of older people (Fraser et al., 2014; Groot et al., 2021). A specific body of research focuses on the potential benefits of the engagement of persons with dementia in the arts (Fraser et al., 2014; Letrondo et al., 2023; Zeilig et al., 2014). These arts activities take place on a continuum between public arts participation, where the focus lies on artistic outcomes that can be shared with a community or an audience, to art integrated in the everyday care context. Research into these practices focuses on the lived experiences of encounters with the arts or artists and the affects, atmospheres, and relationships that develop as a result thereof as well as the positive effects this has on the well-being of persons with dementia (Kontos, Miller, Mitchell, & Stirling-Twist, 2017; McCormick, 2017). There are some studies addressing the potential impact of such practices in long-term dementia care reaching beyond the individual with dementia. For instance, a study showed that art programs had a positive effect on staff attitudes toward persons with dementia, enabling them to see the capabilities of the person with dementia and consequently the person behind the condition (Windle et al., 2020). Another study shows arts-based interventions can have the effect of relieving some of the experienced burden of care on formal as well as informal caregivers and family members (McManus et al., 2022).

### Boundary Work in Arts and Health

Some studies show positive change as a result of arts-based interventions cannot take place without a supporting environment (Bungay et al., 2021; Dadswell et al., 2024; Huhtinen-Hildén, 2014). This means mediating factors for successful delivery of arts-based interventions not only include the artistic process itself but also the collaborative processes within the care setting, such as trusted working relations and clear practical agreements and preparations (Bungay et al., 2021; Dadswell et al., 2024). The work that takes place in this context has been conceptualized as “boundary work” (Daykin, 2019; de Kock et al., 2024). Personal, relational, and organizational factors at play in the context determine whether arts-based interventions can achieve the desired impact. To our knowledge, only a few studies specifically aimed to investigate collaborative working dynamics between the arts and dementia care sectors (Bungay et al., 2021; Dadswell et al., 2024; de Kock et al., 2024; Huhtinen-Hildén, 2014; Puebla Fortier & Coulter, 2021; Tan, 2020). These scholars show that there is a need for research to understand the context influencing the potential impact of such practices (Huhtinen-Hildén, 2014, p. 226). Furthermore, it is necessary to better understand *how* successful boundary work between the arts and care sectors can be facilitated (de Kock et al., 2024). We aim to fill this gap with this study.

### Health Care Clowning in Dementia Care

In this study, we investigate working mechanisms of successful boundary work in arts and health. We did so in a case of health care clowning in dementia care. Health care clowning in the care for persons with dementia is an emerging professional field focused on enhancing the well-being of those individuals through play, (non-)verbal and embodied engagement (Dunn et al., 2013; Hendriks, 2012; Kontos, Miller, Mitchell, & Stirling-Twist, 2017; Molterer & Hoyer, 2019). Clown practitioners, often trained in both performance arts and different therapeutic techniques, work in long-term care settings to engage residents with dementia, aiming to foster affective encounters (Dunn et al., 2013; Molterer & Hoyer, 2019). They will regularly visit residential wards, often in duos, interacting with residents either in a group or individual settings. Clowning in dementia care has been linked to thinking about personhood in relation to this condition (Hendriks, 2012; Kontos, Miller, & Kontos, 2017). For persons living with dementia, the “self” is increasingly formed through the capacity to sustain relationships with others, and the way in which those persons in their direct environment can foster meaningful relationships and interaction (Hendriks, 2012). The practice of health care clowning recognizes the importance of non-verbal communication, adapting to the cognitive abilities and emotional needs of the residents. Clown artists are trained as it were, to meet the person with dementia “in their world,” using the language that best suits them, whether this be play, music, gesture, or verbal utterances. Studies indicate that health care clowning can thus contribute to the thriving of the self, as well as support the agency of the person with dementia, offering an innovative complement to traditional dementia care (Hendriks, 2012; Kontos, Miller, & Kontos, 2017). Previous studies show the potential benefits and working mechanisms of clown visits for persons with dementia in long-term care facilities (Xu et al., 2023). For instance, clown visits have been associated with a reduction in challenging behaviors, agitation, and negative emotions among residents (Kontos et al., 2016; Low et al., 2013). However, interactions between clowns and residents with dementia do not operate in a vacuum. In this multi-country participatory action research, we explore these contextual factors. Furthermore, we explore insights on how elements of a PAR process such as open dialogue and arts-based research methods can be helpful to foster collaboration among all involved. These incidental findings were constructed, tested, and refined as the project progressed.

## Methodology

The aim of the study was to gain understanding of the contextual factors that influence the impact of clown visits in long-term dementia care. The chosen approach was participatory action research (PAR). PAR is a collaborative approach to inquiry, taking insights from different epistemological traditions such as social constructivism, hermeneutics, and critical theory (Abma et al., 2019). From a social constructivist perspective, PAR seeks to address social issues through active involvement of the community members, patients, and professionals affected by those issues. Knowledge construction is viewed as a social and relational process requiring the co-construction of knowledge and the empowerment of marginalized voices (Abma et al., 2019). From a hermeneutics perspective, PAR emphasizes the importance of experiential knowledge, interpreting and giving meaning to lived experiences. PAR takes from critical theory its emphasis on social structure and power imbalances; it challenges who is allowed to have a voice and critiques a vertical epistemology based on expert–layperson dichotomies (Abma et al., 2019). PAR is characterized by its cyclical nature, where reflection and action occur iteratively in a joint learning process. PAR aims to produce actionable knowledge that leads to meaningful change while fostering mutual learning (i.e., Abma & Groot, 2023; Lindquist-Grantz & Abraczinskas, 2020). In the realm of PAR, researchers frequently employ arts-based methods to delve into the experiential and affective dimensions of participants’ knowledge (Abma et al., 2019). It is considered a rich way to access experiential, “felt,” rather than cognitive, knowing (Leavy, 2017; Visse et al., 2019). Moreover, participatory arts-based methods create opportunities for disseminating research in ways other than academic manuscripts (i.e., Rossitier et al., 2008).

### Context of the Study

The research was conducted within four different care organizations across three countries (the Netherlands, Germany, and Austria). At each location, a dedicated research group (PAR group) was formed, which regularly convened from January 2022 to June 2023. Furthermore, the researchers facilitating the PAR processes in the different countries, formed an international research group which regularly came together online, to exchange knowledge about PAR techniques, share findings from the different PAR groups, and jointly analyze the data.

It is important to note that the PAR meetings were set in slightly different contexts in each research location. For instance, in ZuidOostZorg, the Netherlands, in Haus Angerhof Glienicke/Nordbahn, Germany, and in Maimonides Zentrum, Austria, the clowns taking part in the PAR group had already been visiting that care setting for a few years and were well acquainted with some of the welfare and care staff. However, for the clowns, it was the first time talking more elaborately to family members of residents of the care facilities. In Amsterdam, the Netherlands, the PAR group consisted of only clowns and members of welfare staff. Prior to the PAR project, they had never met or worked together, so the PAR project ran simultaneously to the initiation of clown visits in that location.

### Participants, Methods of Data Generation, and Analysis

The number of participants and their background varied in the local settings. Table 1 gives an overview of participants and activities that took place during the research process. The care locations for the research were identified by the partnered clowning organizations, who already had working relations in these settings. Participants were invited in the different care settings by asking family members and members of care staff who would be interested in taking part in a research project around the impact of clowns in the care for persons with dementia. It was explained to all that the project aimed to gain understanding of the contextual factors that influence the impact of clown visits in long-term dementia care.

**Table 1.** Overview of Participants and Research Activities per Location.

Location	Participants in the PAR Group	Research Activities
Amsta, Sarphatihuis, Amsterdam, the Netherlands	• Well-being staff member (*n* = 1)	7 meetings of 1.5 hours with core research group
	• Clowns (*n* = 2)	- Open dialogue sessions (*n* = 4)
	• Facilitating researchers (*n* = 2)	- Joint analysis/knowledge dissemination sessions (*n* = 3)
ZuidOostZorg, Stellinghaven, Appelscha, the Netherlands	• Well-being manager (*n* = 1)	5 bi-monthly meetings of 2 hours with core research group
	• Well-being staff members (*n* = 2)	- Open dialogue sessions (*n* = 2)
	• Health care staff members (*n* = 2)	- Arts-based research session: tableau vivant (*n* = 1)
	• Family members (*n* = 2)	
	• Clowns (*n* = 2)	- Joint analysis/knowledge dissemination sessions (*n* = 2)
	• Facilitating researchers (*n* = 2)	
Maimonides Zentrum GmbH, Elternheim der IKG, Austria	• Well-being staff member (*n* = 1)	7 bi-monthly meetings of 2–3 hours with core research group
	• Health care staff members (*n* = 3)	- Open dialogue sessions (*n* = 4)
	• Health care manager (*n* = 1)	- Arts-based research session: Lego tableau vivant (*n* = 1)
	• Family member (*n* = 1)	
	• Clowns (*n* = 2)	- Joint analysis/knowledge dissemination sessions (*n* = 2)
	• Facilitating researcher (*n* = 1)	
Haus Angerhof Glienicke/Nordbahn, CASA REHA Altenpflegeheim GmbH, Germany	• Well-being staff members (*n* = 2)	9 bi-monthly meetings of 3–4 hours with core research group
	• Family member (*n* = 1)	- Open dialogue sessions (*n* = 7)
	• Clowns (*n* = 2)	- Arts-based research session: tableau vivant (*n* = 2, at the beginning and end of the project)
	• Facilitating researcher (*n* = 1)	Over the project time, each group member observed at least one clown visit and took field notes (*n* = 3 hours of observation)
International research group	• Independent facilitating researchers from the Netherlands (*n* = 2)	• 2 live sessions (4 hours)
	• Researcher from CliniClowns, the Netherlands (*n* = 1)	
	• Researcher from ROTE NASEN, Germany (*n* = 1)	
	• Researcher from ROTE NASEN, Austria (*n* = 1)	
	• Monitoring, evaluation, and learning coordinator from RED NOSES International (*n* = 1)	• 23 online sessions of 2 hours over the course of the research project
	• Relation manager from CliniClowns, the Netherlands (*n* = 1)	
	• Programme manager from RED NOSES International (*n* = 1)	

In all PAR groups, open dialogue sessions were organized. The open dialogue sessions were different each time, but all PAR groups started with the question: “Now that we are all here, what would we like to discuss and what would we like to learn together?” As it was often the first time clowns, family members and members of staff would take this much time to sit down and talk to each other for the purpose of doing research together, conversations started immediately. The facilitating researchers made sure to leave a lot of free space to just chat about whatever came up, which brought up spontaneous conversations about sharing anecdotes of clown visits and quirks of the residents, as further discussed in the Results section. Other open dialogue sessions would build on these first sessions and become more structured as more themes started to be identified by the groups that needed further exploration, such as the roles everyone plays during clown visits. For this further exploration, the researchers organized sessions in which arts-based research activities took place, such as drawing, mind-mapping, LEGO building, and creating tableaux vivant. Tableau vivant is a drama-based method and works with frozen pictures in time, acted out by groups of people (Tortello, 2004). They offer a way to explore movement and development in relationships between all actors in the tableau. In Germany, the facilitating researcher decided to further explore this theme with the PAR group by using existing video footage of clown visits to dementia care wards. The question asked was: “What do you see?” All PAR group meetings were audio recorded and transcribed.

Over a 2-year time period, 23 online sessions and two live sessions were held with the international research group, consisting of all facilitating researchers and different representatives from the participating clown organizations (Table 1). The team of authors kept a jamboard with all findings and notes from these meetings. Additionally, the Dutch research team organized an interactive face-to-face meeting in the Netherlands, presenting the research findings so far, inviting important stakeholders from local clown- and care organizations and the participants of the Dutch PAR groups (*n* = 32 participants).

The analysis in this study was a cyclical, iterative process which took place within all PAR groups throughout the project and in the online meetings with the international research group. Reflexive thematic analysis (Terry & Hayfield, 2020) was used to synthesize all generated data and transcripts of the PAR sessions. In the PAR groups, for example, the facilitating researcher would send out transcripts from the previous two sessions for the group to read and familiarize themselves with, and then during the next meeting they would jointly construct, test, and refine themes. During every online session with the international research group, the researchers first made themselves familiar with the data by sending each other transcripts, fieldnotes, or photographs of the sessions that took place in their PAR groups. Themes were constructed, tested, and refined during reflexive discussions and critical dialogues between the different researchers. These were then taken back to the PAR groups in the different countries to be reviewed and refined. To explore these themes in detail and deepen their understanding, the PAR groups in the different locations created two movies, a talking board, and a checklist for further collaboration as a form of synthesizing and disseminating their most important learnt lessons during the research process. Finally, the international research team jointly wrote a report to synthesize the findings from all PAR groups (de Kock et al., 2024). The contents of all these dissemination products fed into the content of this article.

### Team

The team of researchers consisted of six white women from European descent. The team members have backgrounds in different disciplines, such as participatory health research, nursing, anthropology, social geography, communication, political science, clowning, and theatre studies.

### Ethical Statement

Our study was approved by the scientific ethics committees of participating health care organizations in the Netherlands: ZuidOostZorg and Amsta. All participants of the PAR groups provided verbal informed consent prior to enrollment in the study. Researchers explained the purpose of the study and the aim for publication in a scientific journal during the first PAR meetings, and participants were given the option *not* to take part in the research project. Consent was asked specifically throughout the project whenever audio was recorded or pictures were being taken. Participants recognizably featured in the pictures in this manuscript gave specific verbal consent for the use of these images.

## Results

This section presents the main insights on *how* contextual factors impact the implementation of an arts-based intervention in a long-term dementia care setting. We have structured this section around the research activities that took place in the PAR process: Open dialogue, arts-based research activities, and action and joint dissemination. Each section is divided into sub-themes, which represent the themes that were identified and refined by the PAR groups during those research activities: *Sharing bibliographical details and anecdotes*, *space for critique*, *roles on the playing field*, and *sharing the right information in the right way*. For each topic, we will give some examples of interactions or events that occurred in the PAR groups.

### Open Dialogue

During the PAR group meetings, open dialogue took place between clowns, family members, and staff of the long-term care facilities. These dialogue sessions allowed for insight into the relational context both during and before the intervention.

#### Sharing Biographical Details and Anecdotes

In the PAR group in Austria and Germany, care staff, family members, and clowns shared biographical information about the residents, for instance, the specific quirks or interests they had in their life. In the following example, a family member told the PAR group that their loved one liked Charlie Chaplin. In a fragment from an audio transcript of a PAR group meeting in Austria, the group discusses what the clowns could do with this information:

Clown 1: We have the biographies now. But if we could discuss them in more detail, that would of course be …

Researcher: Before that, do you want to do it in such a way that you come half an hour beforehand and do a handover?

Care staff: Yes, exactly. That we then discuss it in more detail, that’s really important, like with Charlie Chaplin. You can respond to it so well.

Clown 2: Yes, that’s a point where you can create a connection straight away …

This fragment shows that taking time to talk to each other and share biographical information about the residents helped the clowns in their interactions with the person with dementia. What the participants are also saying is that this is not something that usually happens. They are discussing with each other that it would be nice to have a handover before the clown visit to have time to discuss biographical information of the residents in more detail. This was already a common practice on other wards. In the setting of the aforementioned example in Austria, handovers were a common practice too; however, according to the PAR group, the handovers would not go into much biographical detail.

Clown artists, when they visit the wards and are in costume, never break from their role. This feature of clown play is key to the profession. Breaking from the role for interactions with, for instance, care staff or family members, would make it hard for the artist to step back into their role. So, whenever they meet family members or care staff while on their visit, they will encounter them as their clown persona, leaving little space for those professionals to meet the clown artists “behind the red nose,” as one of the clowns stated: “Sometimes people don’t assume right, that we are human beings. As soon as the nose is on, you are something else. Then for some people you are something that is no longer approachable*.*” In some care organizations, when the handover before or after clown visits is not standard protocol, care staff and family members never actually get to speak to the professional and person behind the mask, the red nose. When that nose is off, they get a chance to meet on the same level and have a professional as well as personal dialogue. The PAR showed how important this is to attune to the person with dementia. Thus, a simple but crucial factor that contributed to an open dialogue included reserving time and space for all involved to get to know each other and meet regularly, in person, masks off. The PAR group in Germany mentioned that this is standard protocol there, showing the other PAR groups that this could be possible when time and resources are managed appropriately.

Taking the time to meet outside of the intervention also ensured that the impact of the intervention could reach beyond the individual with dementia, to those in their direct surrounding. Staff and family members could share a sense of affection, curiosity toward, and respect for the older persons under their care. Sharing their family members’ particularities and quirks, and also seeing clowns and members of care staff noticing the same things, showed a shared fondness and recognition of the person and fostered a connection among those surrounding them. For instance, a family member who shared the information about her loved one liking Charlie Chaplin later stressed how valuable it had been for her to be able to just talk and share insights about her husband, and for care staff and artists to listen to her: “Of course, it was particularly interesting for me to learn so much and to be able to exchange ideas. So thank you for letting me be there*.*”

Reserving this time to share moments they remembered well, for instance, where they felt particularly touched in either a negative or positive way by the clown’s visits, meant all PAR group members could share in the joy or challenges of those moments, and find out they were working toward the same goal: ensuring the best quality of care for the person with dementia. This is illustrated in the following conversation, a fragment from the PAR group in Austria, where a staff member and a clown realize the similarities in their approach to a resident in the ward:

Family member: Yes, the ladies (care staff) also motivate him very often and dance with him ...

Wellbeing staff: That’s the kind of everyday activity that we often have on the dementia ward, where we always greet everyone in their own way with “give five” or something else or just dancing …

Care staff: We’ve just noticed that we work well together—you (the clowns) and us …

Researcher: That’s a super combination.

Clown: Yes, totally.

#### Space for Critique

There were also moments during the PAR meetings where more critical attitudes toward each other were expressed. In the following fragment, which stems from the audio transcript of the first meeting of the PAR group at Amsta, the Netherlands, a member of the well-being staff talks about the feedback she received from colleagues when she told them there would be clown visits introduced to their ward. She also discloses that she is apprehensive of clowns herself:

Wellbeing staff member: The feedback was that older persons were not such fans of it (clowns). So, I do like to be there.

Clown 1: Is that the reaction you got from people? From the department?

Wellbeing staff member: Yes … that was the feedback. Also, they said: my children used to be scared to death of clowns. I thought that made a lot of sense when I heard that, I think yes, my kids really had nightmares. So we have a different view of clowns, I guess. So, I also understand that an older person could be scared of that in the same way … which is why I do try very much to prepare people and understand the preconditions. That’s kind of what I’m doing here.

When reading this quote, it is important to remember that this particular PAR group was formed in an institution where the clowns would be newly introduced; hence, this member of staff had no experience of clown visits yet. This fragment shows that the staff member feels she and her colleagues need to protect their residents. She wants to know exactly what can be expected of the clowns and how the clown visits will take place. She also explains her preconceived ideas about clowns and her apprehension.

A few months later, after the clowns had been visiting her ward several times, the artists and the member of staff reflected on the effect of the conversation they had during that first meeting in the fourth PAR group meeting:

Well-being staff member: I was so pleasantly surprised that you recognized that there is resistance. It has the effect of a lid, when it is released, your tension fades. My resistance was almost gone before you came. Recognizing that there is resistance worked very well for me.

Clown: It’s not something we normally do, usually only an appointment is made with the management. We have no idea if it’s the first, second or third time that clowns come. So this [PAR study] is actually a very special project in that respect. I am also thinking what would have happened if we had never had that conversation.

The clowns and the member of staff agreed that this first meeting was beneficial for the collaboration later on. Her being able to express her apprehension, ask any questions she had, and the clowns listening to this, just being able to talk about it, had the effect of her resistance resolving almost instantly. As the clown states here, in her experience, clowns were rarely given the time to properly meet, sit down, and talk to the care staff, and these conversations would normally not take place.

In ZuidOostZorg, the other research location in the Netherlands, the health care staff had more experience working together with clowns. In the PAR group, they elaborated on how clown visits could be difficult and confronting for some family members. One of the staff members stated:

For instance, we have a man who has always been a professor and his family are very impressed by his intellect. So, that is also something you encounter, like, “you cannot, with such an important professor, that is not possible with clowns, right?” There are people who think that their parent is this special person. And of course, he is, but in the meantime, this has nothing to do with how you performed in life. Sometimes this is worse for the family than for the resident.

Family members and members of staff were sometimes apprehensive: could a character like the clown possibly match with their high regard of their loved ones or clients? As a well-being staff member stated: “I am very protective over my residents, I don’t want anyone making fun of them or treating them like children*.*” In the PAR meetings, the participants discussed the different preconceptions about clowns, which they had previously picked up from other staff members or which they thought themselves before encountering health care clowns: “Something for children,” “only there to make fun of you,” and “a silly, disrespectful character from the circus.” All members of the PAR groups felt that these preconceptions or fears might have been incorrect but needed to be taken seriously and addressed. Clowns stated it was valuable for them to know what care staff and family members could think about clowns, as often there seemed to be non-verbal signs of resistance. As one of the clowns in Amsta, the Netherlands, explains:

… what I always find tricky is health care staff … like last time, we were towards the end of our visit and somebody (a member of staff) came in and said “I am the third clown.” And they went to the kitchen and she was talking very loudly to their colleague, while we were working at the tables. Then something really happens … what I would need is to know what they actually think, this has an influence on me in that moment. I think that is a shame, I would like it if that person turned around and looked for a while, at what is actually happening in that moment.

The clown, in this fragment, describes this encounter with a member of staff affects the way she can perform her job in a negative way. She picks up on a sense of resistance, as the person starts to loudly speak over her and perform her duties in the kitchen, which is distracting for the clown trying to establish contact with the residents. However, the incident was never actually spoken about by this clown and the member of staff. Having time to meet and speak to staff outside of the clown visit might resolve this issue.

These interactions discussing more negative attitudes and preconceived ideas about clown visits could take place within this project, because researchers stressed that talking to each other and working together on the PAR project was to *jointly investigate* clown visits and the way they fitted in the context of the long-term care facility. The researchers specifically invited the participants to have an open, curious, critical attitude, share their true feelings, and put anything that was bothering them or that they had heard from colleagues or family members on the table, as this would contribute to gaining the best research results and having real, honest conversations. One of the representatives from the clown organization reflected in a meeting with the international research group:

I think … the question is always about impact, show us the impact. Show the proof and people will be convinced. But actually, I think it is about, if we want to have more impact, it is not about convincing people that it works, but about listening. Really listening to how they feel about us and realizing that as clowns, and clown organizations, we have to work with this reality that people might be scared or have reservations about us. They just want to be heard, we need to just listen to them and have an open conversation.

Throughout the PAR project, the member of staff from Amsta previously quoted grew to trust the clowns, and even said she became one of their biggest fans, insisting that the clown visits would continue after the research project finished. Undertaking the research project simultaneously to the implementation process, as well as collaborating with institutions where clowns had been visiting for longer, was identified as valuable by all participants in the PAR groups; it allowed investigating the integration of the clowns in the care context instead of evaluating or judging each other.

### Arts-Based Research Activities

#### Roles on the Playing Field

Some of the PAR groups investigated the roles each person plays during the clown visits through different arts-based research activities, such as tableau vivant. During the tableau vivant session, different members in the PAR group “stood in for” persons present on the work floor during a clown visit. The PAR group members got a role assigned to them, and positioned themselves in the space, purely based on intuition and where they thought the person they were representing had to be during the clown visit. The researcher leading the session moved the persons around in the space, to try and feel how this would change the dynamic of the clown visit. Questions asked to all on the playing field included: “How do you feel in this position? Do you feel good or bad? Would you want to move, or does somebody else need to move?” The researcher constantly encouraged the participants not to overthink but to feel how they felt in the room. This was the basis for a discussion.

**Figure 1.**
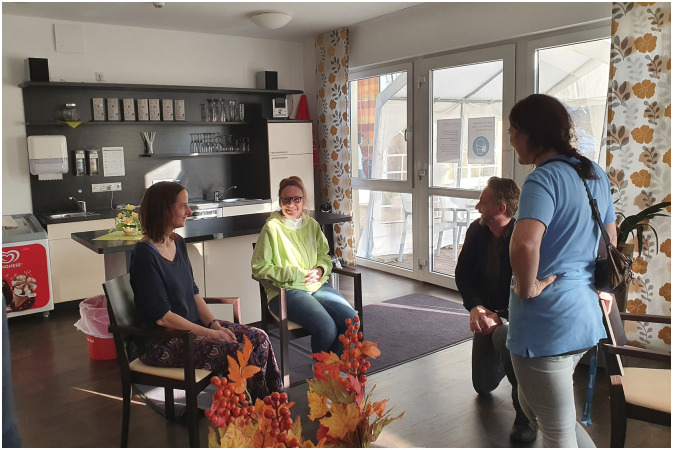
Tableau vivant in Germany: the family member next to the resident, both sat in chairs. The clown entering on the right, on his knees.

**Figure 2.**
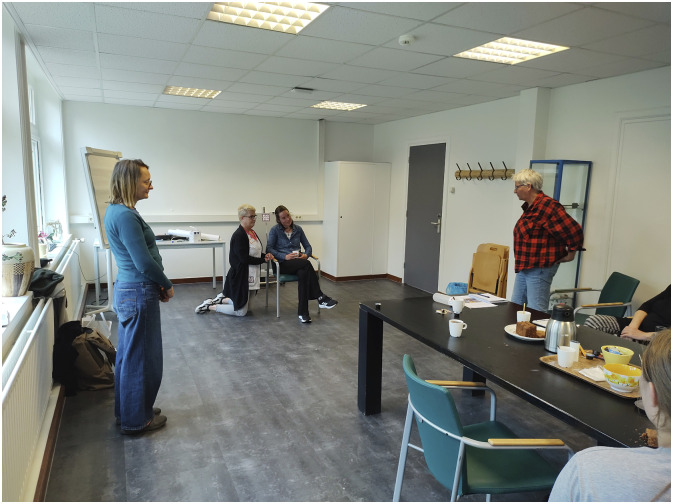
Tableau vivant in the Netherlands: staff member close to the person with dementia, sitting in the chair. The clown entering the room on the right, staying at a distance at first.

Two scenarios unfolded during the tableau vivant sessions in the two PAR groups: The person who was asked to stand in for the resident with dementia first sat down in a chair. Then a member of staff positioned themselves in the room. In Germany, this staff member placed themselves in the far corner, so as not to disturb the clown play. Another person, standing in for a family member, placed themselves next to the resident (Figures 1 and 2). In the Netherlands, the member of staff positioned themselves next to the person with dementia. Then, the person standing in for the clown was asked to step into the room. The clown stayed a little bit at a distance at first, scanning the room. When the person standing in for the person with dementia in the Netherlands was asked how they felt toward the clown, they expressed:

I feel like, what is going to happen? Are you coming to me, or am I coming to you? (Then to the member of staff) I do like that you are with me. That we go together, something like that*.*

Both in Germany and in the Netherlands, the people standing in for the person with dementia experienced that they felt they wanted the person representing a staff member or a family member to stay close to them, or at least within their sight, to feel safe and supported when the clown, someone new and unfamiliar, was approaching.

In Germany, the staff member positioned herself behind the resident in the far corner. When asking her why she was standing there, she replied:

I don’t want to intrude on the visit, but I always love to watch the clowns play. That’s why I usually stand by the door. I feel like it is the clown’s show now and they need the space. I feel I should rather stay in the background and I am not sure how far I am allowed to join in since I am not the one who is visited by the clowns.

The PAR group in Germany discovered that this might be counterproductive: “I feel like I am being watched,” stated one of the clowns. It made her feel less able to be free in her interaction with the resident. The member of staff looking in from a distance felt more like surveilling. The research group concluded that it would be much more productive for the staff member to be closer to the resident and more involved in the clown play. They figured open dialogue about expectations during the clown visit could solve this issue.

The researcher facilitating the session tableau vivant session in the Netherlands then gave a new insight, as the following conversation illustrates:

Researcher: This is a very nice scenario, but I have heard in sessions here, that the reality is often different.

Clown: Yes, it is even sometimes like, there are the clowns, well, have fun! And everyone leaves. Then there you are with the two of you.

The researcher gave the assignment to the person standing in for the member of staff to pretend not to be so fond of clown visits. Then, the following interaction occurred: The researcher asks the person standing in for the member of staff: “What is now your impulse? You are not so fond of clowns, and they enter. What happens now?” The person standing in for the member of staff says she would move to the corner of the room, and does so in that moment. Then the researcher checks with the person standing in for the clown: “And what does that evoke in you?” The clown replies that she feels small, insecure, and rejected. The researcher instructs the clown to show this in her physique, and she makes herself very small in the space. Then the researcher turns to the person standing in for the resident and asks her: “Where does your attention go to now?” The resident replies that she is busy with keeping track of the member of staff who has just moved away from her. When showing what this looks like, she turns away from the clown, toward the corner of the room where the member of staff is standing. The clown then states that she feels this as a rejection; the resident is breaking the connection. However, the resident replies, “It is not that I do not like you”; instead, she continues to explain, all her attention goes to the member of staff: “Look, if the member of staff is next to me, then it can just be nice, together … I feel safe … then suddenly something happens, then I think, oh, I like that.”

What this interaction shows is that this particular person, when standing in for the resident, needed the member of staff to be next to her in order to be able to interact with the clown. The clown explains that she feels like she failed when the member of staff leaves and seems to reject her. She then also feels like the resident does not actually want to interact with her. However, this turns out to be a false assumption, as the person standing in for the resident explains that she simply has her focus on the member of staff, a trusted person for her, and therefore, she does not even consciously notice the clown entering. The research group in Germany also looked at video material of clown visits together, and saw that one of the residents was constantly turning toward the back of the room, where a member of staff was watching the clown play; she also seemed to constantly be aware of the presence of this person, which made her turn away from the clown and unable to fully interact with the artist. What the participants concluded from these sessions and observations in both the Netherlands and Germany was that the role of the member of staff or the family member supporting the person with dementia was crucial. In this case, for the resident to be able to interact with the clown, they needed the other person in the room to be in their sight and involved in the interaction with the clown *with* them.

The sessions unveiled that the roles everyone present in the room plays during the clown visit affect the impact thereof. Furthermore, the research activities provided the participants in the PAR groups with the insight that if all roles on the work floor align and are clear to everyone, then the clown visit can reach beyond the impact on the resident and also include giving positive experiences to any present family members or members of staff. Moreover, the tableau vivant sessions ensured understanding, connection, and trust between the staff, family members, and artists. Before, the member of staff stating she would look on from a distance did not even know that this meant that she could be affecting the clown visit in a negative way. The tableau vivant session helped to uncover unconscious and sometimes wrong assumptions the different people in the room might have about each other and each other’s roles and expectations. They could be doing a counterproductive thing, without realizing it.

### Action and Joint Dissemination

The PAR project facilitated changes in the behavior and interactions of clowns and PAR group participants during subsequent clown visits. This became clear during discussions of the PAR groups. The group members narrated: Clowns realized the potential to actively involve other individuals present in their visits. They began experimenting with engaging staff members through specific questions, which positively impacted their interactions with residents.

Furthermore, the PAR groups sparked discussions about the essential information needed for various stakeholders when clowns visited new care organizations: “The organization or we as clowns need to have conversations with people about what is expected from them when we visit. Staff and family need to feel safe, so they need the right information. What are we doing here? Who are we? What is expected of them when we are here?” stated one of the clowns from the Netherlands during an open dialogue session.

#### Sharing the Right Information in the Right Way

Based on their joint insights and lessons learned, the research groups all developed different prototypes of tools to enhance collaboration between clowns and health care staff. One group created a “Praatplaat” (talking sheet) with a set of cards designed to stimulate conversations among clowns, health care staff, and family members. The other group produced short video clips to provide essential information to health care staff and family members about clowns in dementia care. These videos covered who the clowns are “behind the red nose,” their goals and working methods, and what is expected of residents, health care staff, and family members during clown visits. Additionally, the PAR group in Germany developed a checklist for collaboration, outlining the requirements for successful partnerships with health care organizations. This toolkit serves as a comprehensive guide for fostering effective collaboration and ensuring all parties involved understand their roles and expectations.

Reflecting on the project, participants noted increased awareness and attention to the process. One participant remarked, “Yes, more aware. More aware of the process … now you look much more at the whole process we have gone through together. And also, when a new client comes, you are already alert about how the family might react.” Through continuous dialogue and problem-solving, new opportunities for collaboration emerged. For example, a participant shared:

Initially, some staff said no, no, no, this cannot happen here. We engaged in discussions, and now the door is open again, and there is cooperation. What we did in this whole process, open dialogue, we approached it that way, and it worked.

Moreover, the project influenced training and methodologies for clowns, incorporating the importance of working together well with staff and family members. One of the members of the research team in the Netherlands, also a clown, noted:

I now teach new clowns about this matter, and sometimes an experienced clown might think it’s unnecessary, but I emphasize that it is necessary. It is crucial for our profession to be conscious and act accordingly. Training has become a part of it, and our methodology has been revised.

## Discussion

This study focused on contextual factors influencing the potential impact of health care clowning for persons with dementia, which were explored using a PAR design in order to learn by doing and bring about change in practice. The presented findings demonstrate that PAR, specifically through activities inherent to the PAR process such as open dialogue and arts-based research methods, provided a way to open up a communicative space. Previous studies have shown that open dialogue can provide a way to bridge the gap between system world and life-world (Woelders & Abma, 2019). Wicks and Reason (2009) identify creating communicative spaces and being aware of any obstacles that get in the way of dialogue as a principal task of facilitating participatory research processes. Establishing a communicative space requires a safe environment where participants feel confident that their views will not be used against them. Bergold and Thomas (2012) emphasize the importance of “safe spaces” where individuals can disclose personal views and opinions without fear of repercussion. This openness is crucial for the process of knowledge production, as it allows for the discovery of new aspects of the subject under study and the articulation of different perspectives (Bergold & Thomas, 2012). Inherent principles of the PAR process as described in this study, such as the engagement of multiple stakeholders (clowns, family members, care staff) throughout the project, enhance the relevance of the research, as those involved define the kind of knowledge they need to improve their practice, bringing various perspectives to the fore and creating co-ownership of the knowledge generated (Abma et al., 2017). In this way, the creation of communicative space leads to new insights, learning experiences, and direct changes to practice. As Abma et al. (2017) note, “impact is created along the way as opposed to occurring merely at the end of a research project” (p. 489). In the case of this study, PAR had a positive influence on successful boundary work between the arts and health care sector.

This study demonstrates how the creation of communicative spaces can promote successful boundary work in arts and health. For successful implementation of arts-based interventions in dementia care, focusing on the execution of the intervention itself is not enough. In previous studies, we found that boundary work in arts and health may be a challenging and complex process. Boundary work in that sense literally meant something that takes effort and grit, and requires extra time and resources from all involved (de Kock et al., 2023). This current study aligns with the work of Daykin (2019) emphasizing the need to explore the positive dimensions of boundary work. By focusing on the potential for artistic professionals to successfully collaborate in various fields, groups, and specialties, boundary work becomes a tool for overcoming divisions, amplifying marginalized voices and fostering innovative thinking about shared complex problems. Examining the practices surrounding arts in health and well-being through this lens enriches our understanding of transformational change and its prerequisites in diverse settings (Daykin, 2019). The findings presented in this study show that boundary work, especially personal and relational processes surrounding the arts-intervention in daily practice, can be a catalyst for positive change within the intersection of arts and health care.

Any artist working in a health care setting, like the clowns in this study, may be perceived as an outsider; a stranger coming in, acting in a manner that is unexpected and unknown to other professionals and next of kin. As Dadswell et al. (2024) found, “Pre-conceptions may lead to … anxiety, resistance and rejection of ideas amongst care home staff” (p. 7). Therefore, clowning provides an interesting case to investigate dynamics in the collaboration between the arts and health care sector. During this study, the clowns needed to take off their red nose and meet with the care staff and family members as people and professionals “behind the red nose.” Although the clown is literally a character, the same principle may also apply to other professions; when you do not get to know each other and each other’s methods, knowing each other’s preconceived ideas or possible fears and what is expected of each other, there is no way of being able to collaborate properly. The other person becomes a character in a way, surrounded by mystery and causing possible fear and resistance instead of openness and curiosity to work together. Hence, our findings are in line with previous studies (Bungay et al., 2021; Dadswell et al., 2024) and the importance of building trusted working relationships around arts-based interventions in health care settings.

Reflecting on the findings, the study underscores the transformative potential of boundary work. It suggests that a boundary creates a possibility to look at oneself through the eyes of other worlds, leading to changes in practices (Akkerman & Bakker, 2011). Drawing parallels with the concept of boundary-crossing laboratories, our study suggests that the PAR process itself can act as such a laboratory. By bringing people from different systems of activity together to discuss and work on shared problems at the boundary, researchers can serve as mirrors, confronting individuals with the problems they share (Akkerman & Bakker, 2011). This study served as a boundary-crossing laboratory, facilitating interactions and discussions that contribute to transformative changes within the health care landscape, such as the introduction of new ways of educating clowns, and choosing to facilitate open dialogue between clowns and care staff instead of merely providing information about clown visits.

Finally, this study raises important questions about the introduction of arts in health care settings, specifically whether PAR, as introduced in the different settings in this study, is always necessary or if, for instance, a process facilitator from within or from outside of the involved organizations could also achieve similar outcomes. If so, what characteristics, roles, and responsibilities should this facilitator possess? Is it conceivable that such facilitation is particularly crucial when an organization is working with artists for the first time? Once the initial process has been successfully navigated, subsequent collaborations are likely to proceed more smoothly, at times still using some PAR techniques, such as organizing open dialogue sessions when there are any changes in circumstances that cause collaboration to temporarily run less smoothly. This suggests that while PAR provides a ray of collaborative techniques for integrating arts into health care, alternative facilitation approaches may also be effective, especially as organizations become more experienced in these interdisciplinary collaborations.

## Conclusion

In conclusion, this study highlights the role of PAR as a catalyst for boundary work in the context of arts-based interventions in health care. The findings indicate that PAR facilitates the creation of a communicative space, promoting good collaboration that bridges the arts and health sectors. By engaging multiple stakeholders and fostering open dialogue, PAR instigates meaningful changes in practice during the research process itself. The research activities inherent to a PAR process enable diverse participants from the arts and health care fields to collaborate effectively, share knowledge, and co-create solutions, ultimately enhancing the quality of care for individuals with dementia. Future studies should explore the potential of process facilitators who may not be independent researchers, but managers, artists, or care staff from the different involved stakeholders, in achieving similar outcomes. By recognizing and harnessing the immediate impact of PAR, it can become possible to integrate artistic practices in health care and foster innovative, collaborative environments that benefit both care recipients and providers.

### Strengths and Limitations

The study’s strengths lie in its long duration, large number of participants, and the use of diverse participatory methods. Moreover, conducted across different countries, the research encompassed various cultural backgrounds and languages, thereby enhancing its generalizability. The active participation of clowns, family members, care staff, and welfare staff throughout the research process provided comprehensive and thick descriptions. However, there are notable limitations. The focus on clowning means the findings cannot be directly extended to all arts-based interventions. Additionally, no persons with dementia were directly involved in the research. Although the researchers attempted to “stand in” for these individuals during the tableau vivant sessions, and family members and care staff were very closely involved and observant of the persons with dementia under their care, it is important to acknowledge that the experiences and perceptions of persons with dementia may differ significantly. It is essential to recognize individual differences in the way certain situations are perceived and experienced and to always closely observe signs of discomfort, especially given the varied backgrounds and experiences of all persons with dementia.
